# Short sickness absence and subsequent sickness absence due to mental disorders - a follow-up study among municipal employees

**DOI:** 10.1186/s12889-016-3951-7

**Published:** 2017-01-05

**Authors:** Hilla Sumanen, Olli Pietiläinen, Eero Lahelma, Ossi Rahkonen

**Affiliations:** Department of Public Health, University of Helsinki, PO Box 20, Tukholmankatu 8B, FIN-00290 Helsinki, Finland

## Abstract

**Background:**

Mental disorders are common diagnostic causes for longer sickness absence and disability retirement in OECD-countries. Short sickness absence spells are also common, and neither trivial for health and work ability. We studied how prior short sickness absence spells and days are associated with subsequent longer sickness absence due to mental disorders in two age-groups of municipal employees during a 2-, 5- and 9-year follow-up.

**Methods:**

The analyses covered 20–34 and 35–49-year-old employees of the City of Helsinki in 2004. Those with prior ≥14 day sickness absence in 2002, 2003 or 2004 were excluded. Women and men were pooled together. Short, 1–13-day sickness absence spells and days were calculated per the actual time of employment during 2004. Logistic regression analysis was used to calculate odds ratios (OR) and their 95% confidence intervals (CI) for the subsequent long (≥14 days) sickness absence due to mental disorders during three follow-ups.

**Results:**

The risk for long sickness absence due to mental disorders increased with increasing amount of short sickness absence spells and days. 3 or more short sickness absence spells and 8–14 sickness absence days from short spells in 2004 were strongly associated with subsequent long sickness absence in all three follow-ups. The associations were strongest for the 2-year follow-up; the younger employees tended to have higher risks than the older ones.

**Conclusions:**

Three spells or 8 days of short sickness absence per year constitutes a high risk for subsequent long sickness absence due to mental disorders and preventive measures should be considered.

## Background

Mental disorders challenge work ability and efforts for prolonged working careers. Mental disorders contribute increasingly to sickness absence from work in OECD countries. Also in Finland, mental disorders are the most common diagnostic cause for long sickness absence and disability retirement alongside with musculoskeletal disorders [1, 2]. Prevention of mental disorders and supporting those with high risk at an early stage is crucial for successful prevention of work disability and the related financial costs.

Mental disorders typically take time to develop and show early symptoms, and short sickness absence may be an indicator of such underlying disease. In this study short sickness absence refers to spells lasting 1–13 days and long to ≥14 days, but the definitions vary in previous studies. Overall the role of shorter sickness absence as a health indicator is likely limited, as shorter sickness absence may, to some extent, reflect employees’ perception of their health and sickness absence behaviour rather than an actual disease [3, 4]. Also, because the diagnoses are typically available only for sickness absence spells lasting over two weeks, the reasons for shorter absences are less well known. However, according to our previous study, especially young employees have a greater amount of 1–14 days sickness absence spells than older employees, who in turn have a greater amount of >14 day spells [5]. In a previous Finnish study [6] on middle-aged employees both 1–3 day and 4–14 day sickness absence spells predicted subsequent sickness absence lasting over two weeks. The risk of such sickness absence increased linearly with the number of prior shorter spells among both women and men even after adjustments for occupational class, working conditions and health behaviours. Overall studies have shown that shorter sickness absence spells, especially frequent, predict longer sickness absence, suggesting that shorter spells are not trivial for subsequent ill-health and work disability [6, 7].

Our study aims to add evidence on how short sickness absence, spells and days, predict subsequent long sickness absence due to mental disorders among 20–34 and 35-49-year-old employees and over different lengths of follow-ups. We examined whether short, 1–13 day sickness absence predicts subsequent long sickness absence due to mental disorders over a follow-up of 2, 5 and 9 years among the employees of the City of Helsinki.

## Methods

### Data

This study is a part of the Helsinki Health Study on health and well-being among employees of the City of Helsinki, Finland [8]. The City of Helsinki is the capital of Finland with approximately 600 000 inhabitants. The City of Helsinki is the largest employer in Finland with approximately 40 000 employees and it operates mainly in health care, education, social welfare services, public transport, culture, construction and technical services.

All employees of the City of Helsinki are covered by the same personnel administration, registration systems and policies, which largely remained during the follow-up periods [8]. All employees working for the municipality have equal access to occupational health care services. According to the guidelines of the City of Helsinki, employees can take self-certified sickness absence up to three days with the permission of their supervisor. A qualified nurse can certify a sickness absence spell up to seven days, after which certification from a medical doctor is mandatory.

The personnel register of the City of Helsinki was used to obtain individual-level information on the employees’ socio-demographic background and sickness absence in 2004 in an accuracy of 1 day. Consecutive and overlapping spells were combined. Data on sickness absence due to medically certified mental disorders (ICD-10 codes F00-F99) lasting ≥14 days with diagnostic information from 2004 to 2013 were collected from the registers of the Social Insurance Institution of Finland. Educational level was obtained from the Statistics Finland register of completed education and degrees, and was linked to the City of Helsinki personnel registers. Education was classified into four levels: higher education (a Master’s or a doctor’s degree), upper secondary (a Bachelor’s degree from a university or institution of applied sciences), lower secondary (upper-secondary school, vocational school) and basic education (comprehensive school). Descriptive statistics in Table 1 show that lower and upper secondary educations were most common educational levels in both age-groups.

**Table 1:** Descriptive statistics for the study population

	20–34	35–49
*N*	8027	11663
Working years	5156	10057
Women/Men %	73/27	73/27
Educational level in 2004, %		
Higher	10.4	17.1
Upper secondary	27.3	32.1
Lower secondary	50.7	38.5
Basic	11.7	12.3
Short sickness absence spells in 2004. % (/working-years)		
0	40	38.9
1	9.3	16.6
2	10.3	13.1
3 or more	40.4	31.4
Short sickness absence days in 2004% (/working-years)		
0	40	38.9
1–7	25.5	33.3
8–14	14.5	14.1
15–29	12.6	9.7
≥ 30	7.4	4.0
Long sickness absence due to mental disorders, %		
2-Year follow-up	5	5.3
5-Year follow-up	10.7	11.3
9 Year follow-up	16.3	16.7

Two age-groups were used based on previous research on short sickness absence [5]. 50-years-old and older were excluded due to possible retirement during the follow-up, which may affect to the registration of long sickness absence. We excluded part-time employees working for less than 28 h per week because we concentrated to those who are able to work full-time and part-time employees’ short sickness absences might not be always fully registered to the personnel system. Those with longer, ≥14 days sickness absence in 2002, 2003 or 2004 were also excluded (*n* = 1925 among 20–34-year-olds (which is 19% of all 20–34-year-old full-time employees of the City of Helsinki) and *n* = 4865 among 35–49-year-olds (29%, respectively)).

### Statistical methods

Short sickness absence spells and days were calculated per years in employment, i.e., the actual time of employment during 2004. Logistic regression analysis was used to calculate odds ratios (OR) and their 95% confidence intervals (CI) for the subsequent sickness absence due to mental disorders during 2-year (end of 2006), 5-year (end of 2009) and 9-year (end of 2013) follow-ups. Those without short sickness absence spells or days served as reference group (OR = 1.0). As the associations were similar among women and men in the preliminary analyses and no statistically significant gender interactions were detected, the final analyses were carried out using pooled data, adjusting for gender as a covariate (Model 1). In Model 2, in addition, education was adjusted for. All analyses were conducted using IBM SPSS Statistics version 22.

## Results

Forty percent in both age-groups had no short sickness absence spells or days in 2004 (Table 1). Twenty percent of the 20–34-year-old and less than a third of the 35–49-year-old employees had 1–2 short sickness absence spells. Forty percent of the 20–34-year-olds had 3 or more sickness absence spells, in the older group the percentage was lower. Examining sickness absence days, the younger age-group differed from the older group: a smaller proportion had a maximum of 7 absence days, and a larger proportion had over two weeks long absence. During the 2-, 5- and 9-year follow-up, both age-groups had very similar amounts of long sickness absence spells due to mental disorders.

Logistic regression analysis, adjusted for gender (Model 1), showed that 1 short, i.e., 1–13 day sickness absence spell in a working-year was associated with subsequent long sickness absence due to mental disorders only among 20–34-year-old employees during 2- and 5-year follow-ups (Table 2). However, 2 spells per working-year was in all cases associated with sickness absence due to mental disorders irrespective of the length of follow-up and most strongly during the 2-year follow-up among 20–34-year-olds (OR 2.15, CI 1.48, 3.12). For 3 or more short sickness absence spells the ORs for long sickness absence were higher for all three follow-ups, being highest during the 2-year follow-up (OR 3.01, CI 2.33, 3.89 among 20–34-year-olds and OR 2.80, CI 2.29, 3.42 among 35–49-year-olds). The ORs were somewhat higher among 35–49-year-olds than the younger group during the 5-year (OR 2.64, CI 2.29, 3.04 among 35–49-year-olds and OR 2.50, CI 2.11, 2.96 among 20–34-year-olds) and 9-year follow-ups (OR 2.51, CI 2.23, 2.83 and OR 2.32, CI 2.02, 2.66 respectively). Overall the ORs were broadly similar among the older age-group irrespective of the length of the follow-up. Further adjustment for education (Model 2) attenuated the estimates only slightly.

**Table 2:** Short sickness absence spells/working days and subsequent long mental sickness absence during 2-, 5- and 9-year follow-up among 20–34 and 35–49-year-old employees of the City of Helsinki. Odds ratios (OR) from logistic regression analyses and their 95% confidence intervals (CI)

	Model 1		Model 2	
Age-group	20–34	35–49	20–34	35–49
	OR (95% CI)	OR (95% CI)	OR (95% CI)	OR (95% CI)
2-year follow-up, short sa spells in 2004				
0	1	1	1	1
1	1.60 (1.05, 2.54)	1.18 (0.89, 1.57)	1.62 (1.06, 2.48)	1.18 (0.89, 1.56)
2	2.15 (1.48, 3.12)	1.34 (1.00, 1.80)	2.17 (1.49, 3.14)	1.33 (0.99, 1.78)
3 or more	3.01 (2.33, 3.89)	2.80 (2.29, 3.42)	2.93 (2.27, 3.79)	2.75 (2.24, 3.37)
5-year follow-up, short sa spells in 2004				
0	1	1	1	1
1	1.43 (1.07, 1.90)	1.12 (0.92, 1.37)	1.45 (1.09, 1.94)	1.11 (0.92, 1.36)
2	1.49 (1.14, 1.95)	1.38 (1.13, 1.68)	1.50 (1.15, 1.97)	1.35 (1.11, 1.66)
3 or more	2.50 (2.11, 2.96)	2.64 (2.29, 3.04)	2.41 (2.03, 2.86)	2.55 (2.21, 2.94)
9-year follow-up, short sa spells in 2004				
0	1	1	1	1
1	1.21 (0.95. 1.54)	1.09 (0.93, 1.28)	1.23 (0.96, 1.56)	1.09 (0.92, 1.28)
2	1.41 (1.13, 1.75)	1.35 (1.14, 1.59)	1.42 (1.14, 1.77)	1.33 (1.13, 1.57)
3 or more	2.32 (2.02, 2.66)	2.51 (2.23, 2.83)	2.24 (1.95, 2.58)	2.46 (2.18, 2.77)

Absence days for short sickness absences were also associated with subsequent sickness absence due to mental disorders (Model 1, Table 3.) 1–7 days sickness absence showed fairly weak associations, but 8–14 days of absence showed stronger associations with long sickness absence, and the highest OR (2.63, CI 1.91, 3.62) was among 20–34-year-olds during 2-year follow-up. The ORs for subsequent long sickness absence due to mental disorders increased with increasing days of absence in 2004 in both age-groups and in all follow-ups. During 5- and 9-year follow-ups the associations were stronger among the 35–49-year-olds for 8–14 and 15–29-days of preceding short sickness absence, but when previous short sickness absence days were 30 or more, 20–34-year-olds showed stronger associations. Overall the associations were strongest in shortest, 2 year follow-up with preceding 30 or more sickness absence days among 20–34-year-olds (OR 5.15, CI 3.70, 7.16). Adjusting for education (Model 2) attenuated the associations only slightly in all age-groups and length of follow-ups.

**Table 3:** Sickness absence days/working days (from short sickness absence spells) and subsequent long mental sickness absence during 2-, 5- and 9-year follow-up among 20–34 and 35–49-year-old employees of the City of Helsinki. Odds ratios (OR) from logistic regression analyses and their 95% confidence intervals (CI)

	Model 1		Model 2	
Age-group	20–34	35–49	20–34	35–49
	OR (95% CI)	OR (95% CI)	OR (95% CI)	OR (95% CI)
2-year follow-up, short sa days in 2004				
0	1	1	1	1
1–7	1.63 (1.19, 2.21)	1.29 (1.03, 1.62)	1.63 (1.20, 2.22)	1.29 (1.03, 1.62)
8–14	2.63 (1.91, 3.62)	2.44 (1.92, 3.12)	2.61 (1.89, 3.59)	2.43 (1.90, 3.10)
15–29	3.38 (2.47, 4.62)	3.17 (2.45, 4.08)	3.32 (2.43, 4.56)	3.14 (2.42, 4.06)
≥ 30	5.15 (3.70, 7.16)	4.44 (3.25, 6.08)	4.99 (3.57, 6.99)	4.38 (3.18, 6.03)
5-year follow-up, short sa days in 2004				
0	1	1	1	1
1–7	1.37 (1.11, 1.68)	1.24 (1.06, 1.44)	1.38 (1.12, 1.70)	1.23 (1.05, 1.43)
8–14	2.01 (1.61, 2.51)	2.42 (2.04, 2.87)	1.98 (1.58, 2.47)	2.37 (2.00, 2.82)
15–29	2.92 (2.36, 3.62)	3.20 (2.67, 3.84)	2.83 (2.28, 3.51)	3.11 (2.58, 3.74)
≥ 30	4.21 (3.33, 5.33)	3.57 (2.80, 4.56)	3.96 (3.11, 5.03)	3.41 (2.66, 4.38)
9-year follow-up, short sa days in 2004				
0	1	1	1	1
1–7	1.26 (1.06, 1.49)	1.24 (1.09, 1.40)	1.27 (1.07, 1.50)	1.23 (1.08, 1.40)
8–14	1.87 (1.56, 2.25)	2.18 (1.88, 2.53)	1.85 (1.54, 2.22)	2.15 (1.86, 2.50)
15–29	2.73 (2.28, 3.27)	3.15 (2.69, 3.69)	2.65 (2.21, 3.17)	3.09 (2.64, 3.63)
≥ 30	3.92 (3.19, 4.81)	3.40 (2.73, 4.22)	3.70 (3.01, 4.56)	3.30 (2.65, 4.11)

Model 1: Gender adjusted

Model 2: Gender and educational level (basic, lower secondary, upper secondary, higher education) adjusted

## Discussion

This study examined whether short 1–13 day sickness absence predicts subsequent long sickness absence due to mental disorders over a follow-up of 2, 5 and 9 years. Our main results were: 1) The risk for long sickness absence due to mental disorders increased with increasing amount of short sickness absence spells. 2) More sickness absence days from short spells strengthened the associations for subsequent long sickness absence due to mental disorders. An association could be found starting from 1 to 7 days absence. 3) The associations were strongest for the 2-year follow-up, and weakened slightly over time. 4) 20–34-year-olds tended to have higher risks for subsequent long sickness absence during the 2-year follow-up than 35–49-year-olds, but during 5- and 9-year follow-ups the age-differences were weak or reverse. 5) Educational level had only slightly impact on the association of short sickness absence and subsequent long sickness absence due to mental disorders.

The employees in this study could be divided roughly to two main groups: a large proportion had no sickness absence at all in 2004, and another large proportion had many short sickness absences and with that a high risk for subsequent long sickness absence due to mental disorders. We excluded those who had a sickness absence spells of two weeks or longer in 2002, 2003 or 2004, i.e., those with even higher amounts of sickness absence. The exclusion was made to ensure that the employees were without such ill-health or diseases which require long sickness absences from work. However, even among the employees with only shorter sickness absence we could identify a group at risk of work disability.

Studies on the consequences of short sickness absence spells and days have been rare, and the definitions varies by each study. However, the previous results show that there is an association between shorter sickness absence and subsequent longer sickness absence [6, 7]. A Swedish study [7] with initially healthy cohort of 18–59-year-old employees showed that more than one short sickness absence spell increased the risk of longer sickness absence in 5-year follow-up, even after adjustments for socioeconomic status and mental well-being. A high amount of short sickness absence strengthened the associations more than low amount. A recent Swedish study [9] with a large cohort of young adults found an association between sickness absence in 1993 and subsequent sickness absence from work during 15-year follow-up. In that study there were only sickness allowance days calculated, so the spells less than 15 days were not included. However, the results showed that those who had no and those who had 1–7 sickness allowance days in 1993 had 313 and 567 sickness allowance days during the next 15 years, respectively. Thus, the increase was remarkable, and the later amount of sickness allowance days increased steadily for every increase in length of sickness allowance days in 1993. In summary, similar associations have been found in previous studies and for all-cause subsequent sickness absence. Our study adds evidence on the association for subsequent sickness absence due to mental disorders. Further study is needed to find out if a similar association exists also between short sickness absences and long absences for musculoskeletal diagnoses.

In our study, even one short sickness absence spell per year increased the risk of subsequent long sickness absence due to mental disorders among 20–34-year-olds. However, 3 or more short sickness absence spells per working-year was the point where the associations were consistently strong among both age-groups. In the case of sickness absence days (only short spells), even 1–7 days showed associations, but 8–14 days per working year strengthened the associations for long sickness absence due to mental disorders in all three follow-ups. The risks were highest during 2-year follow-up among both age-groups. This likely suggests that early interventions to prevent work disability, such as support from the supervisors or contact to occupational health care, are needed. Early signs of work disability include repeated short spells and increasing sickness absence [10]. Almost a third of the employees, among younger ones somewhat more, had 3 or more short spells and more than 8 days of sickness absence per working-year. The costs of the sickness absences are high and subsequent long sickness absence due to mental disorders threatens work ability even more and perhaps recurrently [11]. Prevention at an in early stage may save costs in the longer run, as the pathway to early exit from work may initiate with short sickness absence which possibly increases in length and contributes to the risk of work disability and early retirement [10]. Sickness absence due to mental disorders was associated with a 70% increase in the risk of all-cause mortality [12].

The younger employees with short sickness absence tended to have higher risks for subsequent long sickness absence due to mental disorders during the 2-year follow up. Mental disorders appear often at a relatively young age and they are a major diagnostic cause of disability retirement among young persons [13, 14]. In our study the proportion of those who had several short spells or more than 8 days of short sickness absence was highest among 20–34-year-old employees, who should have a good health given their young age. According to previous findings younger persons might have long sickness absence due to mental disorders more often than older employees [15, 16], but overall the age-differences need further studies. Based on the findings discussed, younger employees should be increasingly targeted in the prevention of work disability due to mental disorders.

In prior studies educational level had an impact on the differences in sickness absence, and also for short sickness absence [17–19] but mental disorders do not always show as consistent socioeconomic gradients [20]. In our study, educational level had negligible impacts on subsequent sickness absence due to mental disorders. However, more detailed sensitivity analyses (data not shown) showed that educational groups had very similar amounts of subsequent long sickness absence, except the group with the highest education had less than others, which is in line with a previous study [21].

### Methodological considerations

This study was based on a large number of employees of the City of Helsinki. Information on their sickness absence was drawn from the employer’s registers (short sickness absence in 2004) and from national registers kept by the Social Insurance Institution of Finland (long mental sickness absence during follow-ups). The registers constitute a reliable and comprehensive data source. However, registers lack further information on the participants and their health-related background.

The missing diagnostic information on short sickness absence is a limitation, but in Finland there are no complete data with diagnoses for shorter absences than those in the Social Insurance Institution of Finland’s register, and similar weakness concerns all the Nordic countries with good national registers. As we have full sickness absence records since the employment begins for the City of Helsinki, some of the studied employees had started their employment in the City of Helsinki later than 2002, and they may have prior long absences which we could not exclude. Also, the education level may have risen for some of the employees during the follow-ups, which may slightly affect the accuracy of adjustment.

The participants in our study were municipal employees of a single employer. This has advantages and limitations. The sickness absence and occupational health care procedures are equal for all studied employees and broadly similar across all municipalities in Finland, but may differ from private sector employers. Therefore the results may be generalized with caution to the Finnish municipal sector, but not to the labour force in general.

## Conclusions

Employees whose short sickness absence exceeded 3 spells or 8 days per year had strong risks for subsequent long sickness absence due to mental disorders. The risk was highest during the next few years. Overall a large proportion of employees had 3 or more short sickness absence spells or at least 8 days of sickness absence from those spells. These groups might benefit from preventive measures, especially the younger ones. Early prevention of mental disorders would support employees’ work ability and save high costs for the employer and society.
